# Semi-Supervised Bayesian Classification of Materials with Impact-Echo Signals

**DOI:** 10.3390/s150511528

**Published:** 2015-05-19

**Authors:** Jorge Igual, Addisson Salazar, Gonzalo Safont, Luis Vergara

**Affiliations:** Departamento de Comunicaciones, Universitat Politecnica de Valencia, Camino de Vera s/n, 46022 Valencia, Spain; E-Mails: asalazar@dcom.upv.es (A.S.); gonsaar@upvnet.upv.es (G.S.); lvergara@dcom.upv.es (L.V.)

**Keywords:** impact echo, accelerometers, mixture of Gaussians, semi-supervised Bayes classification

## Abstract

The detection and identification of internal defects in a material require the use of some technology that translates the hidden interior damages into observable signals with different signature-defect correspondences. We apply impact-echo techniques for this purpose. The materials are classified according to their defective status (homogeneous, one defect or multiple defects) and kind of defect (hole or crack, passing through or not). Every specimen is impacted by a hammer, and the spectrum of the propagated wave is recorded. This spectrum is the input data to a Bayesian classifier that is based on the modeling of the conditional probabilities with a mixture of Gaussians. The parameters of the Gaussian mixtures and the class probabilities are estimated using an extended expectation-maximization algorithm. The advantage of our proposal is that it is flexible, since it obtains good results for a wide range of models even under little supervision; e.g., it obtains a harmonic average of precision and recall value of 92.38% given only a 10% supervision ratio. We test the method with real specimens made of aluminum alloy. The results show that the algorithm works very well. This technique could be applied in many industrial problems, such as the optimization of the marble cutting process.

## 1. Introduction

The field of non-destructive testing (NDT) of materials is a wide area, including any technique that extracts information about the condition of a material specimen without altering its physical and/or chemical properties (see, e.g., [1] for a survey of different NDT methods).

Two main elements appear in NDT: sensors and data processing. While sensors are very application dependent and impose practical limits about monitoring resolution, data processing considers general techniques, which may find application in a variety of significantly different NDT problems. From another perspective, sensors are limited by the current sensor technology; meanwhile, data processing is only limited by the required computational resources. On the other hand, non-destructive methods often lead to automatic implementations, thus allowing “on-line” monitoring of large amounts of specimens.

The sensory system poses the essential resolution limits that can be reached to measure the material state. However, large improvements can be achieved in the overall system performance by improving the data processing methods. Although based on general techniques, these methods must take into account the specific context where the NDT is to be applied. Hopefully, once success is demonstrated in that specific context, the method could be extended to other significantly different NDT problems.

In this paper, we present a new classification method, which is specifically oriented to scenarios where some degree of supervision is allowed. The general goal is to classify the specimen under analysis in one of a predefined number of classes. The classifier is trained on the basis of a set of feature vectors previously computed using the same NDT method and sensors. A subset of the whole set is labeled, while the rest is considered of an unknown class. This is termed semi-supervised learning [2] and makes sense in those NDT problems where some selected specimens can be “*a posteriori*” analyzed in a destructive manner, so that the “true” class of the specimen could be known to train the classifier of future specimens incoming to the system.

We apply the proposed data processing in the context of one particular NDT method: impact-echo (IE) [3]. In this technique, a material is impacted with a hammer, which produces an acoustic response that is collected by the sensory system located on the surface of the material. Usually, a set of sensors are distributed across the different sides of the specimen to extract exhaustive information about its inner state. The underlying physics is that of acoustic wave propagation in solids, where different types of waves propagates into the solid and are recorded by properly-selected sensors when they arrive at the surface. The waves are P-wave (normal stress), S-wave (shear stress) and R-wave (surface or Rayleigh). Then, signal processing is performed on the collected signals to extract features that, grouped in a vector form, are the inputs to the automatic classifier subsystem. The feature vector is preprocessed using principal component analysis (PCA) [4]. PCA allows reducing the dimension of the input feature vector while retaining most of the variation in the original data. It is used in many classification problems, including the IE field [5].

IE is a low-resolution technique, which has been extensively applied to monitor the general state of specimens. It has attractive advantages, like low cost, rapid global analysis, deeppenetration and “on-line” processing capability. It is not appropriate for exact localization or characterization of inner defects, where other higher resolution NDT methods, like ultrasonics, are more adequate. However, we will show in the experimental part of this paper that some specific information about the inner state can be obtained by properly defining the targeted classes of the classifier.

Signal processing in IE can be roughly organized into four classes depending on the assumptions considered: time domain, frequency domain, time-frequency domain and machine learning. The first IE works were in the time and frequency domain. Time domain analysis is based on the estimation of successive P-wave arrivals (multiple reflections between the parallel surfaces of a plate) that allows the period and dominant frequency of the waveform to be estimated. In practice, the conditions of an ideal plate are difficult to reach, and thus, a quick interpretation of the results in the time domain is also difficult [6]. In frequency domain analysis, the fast Fourier transform (FFT) is used to obtain the spectrum of the impact-echo signal. The value of the maximum peak frequency in the amplitude spectrum is used to determine the thickness of the plate (see, e.g., [7]).

Spectral analysis of IE signals was improved using time-frequency techniques considering their non-stationarity, *i.e.*, the transient nature of the IE signals. The principal aim was to overcome the problem of noisy signals where the reflections are not clearly distinguished in the spectrum, which shows multiple peaks due to artificial energy added by relatively strong R-waves. This is particularly pronounced in cases of limited dimension specimens. Several time-frequency techniques, such as short-time Fourier transform (STFT) and Hilbert–Huang, have been applied to improve the accuracy in thickness estimation [8,9]. Recently, systematic errors in thickness estimation from IE testing due to near-field effects on the P-wave and R-wave were investigated [10].

The ultimate advances in IE signal processing research came from the field of machine learning and statistical pattern recognition. These methods extract some features from signals of specimens of known classes and use them to train a pattern recognition algorithm that can be used to classify other specimens of an unknown class. Several NDT applications can be suited to this framework, for instance the classification of a material depending on the kind and number of defects, which is the problem addressed in this paper. The degrees of freedom afforded by this framework facilitate multichannel analysis, simultaneous use of features from different domains and the combination of different NDT methods.

Some examples of the combination of IE with other NDT methods to improve the results of defect detection problems are the following: combination with the impulse-response method for identifying delaminations in concrete floor toppings [11] and combination with ultrasonic pulse echo and ground penetrating radar data (GPR) for detecting built-in honeycombing in scale concrete specimens [12].

The machine learning methods most commonly applied in IE signal analysis are based in artificial neural networks (ANN). The problems studied with these methods include: prediction of the concrete compressive strength and thickness of concrete structures [13]; prediction of the internal grouting quality of prestressed ducts [14]; and identification of the pull-off adhesion of the concrete layers in floors on the basis of parameters evaluated on the structural layer surface [15]. Recently, a linear subspace representation of the original features, called the Grassmann manifold, was applied in IE. It was demonstrated that subspace representation could characterize relevant time-frequency distribution patterns and form significant clusters that are separable using a distance [5].

The machine learning method proposed herein has the following advantages compared with the other methods mentioned above: (I) enabling semi-supervised learning (capable of incorporating different proportions of unlabeled and labeled data); this facilitates a quick implementation of the method with a very small sample of specimens, faster than other supervised methods, such as ANN (the difficulties and cost of obtaining labeled data have been extensively studied; see, e.g., [2]); (II) the level of operation of the classification system can be adjusted depending on the percentage of false alarms allowed; (III) the proposed multichannel setup allows mass spectra to be captured from the IE testing experiments that register the differences between defective and homogeneous kinds of materials; (IV) the advantages of a generative model; we obtain posterior probabilities for every class, so this probability can be used in many different ways, not only for basic classification purposes, such as the maximum *a posteriori* (MAP) estimate.

IE has been applied in different types of materials, such as marble, concrete or steel [11,16–18]. In those cases, large blocks are inspected to ascertain the general quality before cutting the material into slabs. This prevents the possibility of accidents during the cutting process, which can deteriorate the machinery and be dangerous to the human operators. It also helps in the setting of the block quality, *i.e.*, in the final price of the material. Training a block classifier is possible in this type of application by selecting a set of blocks where training feature vectors are obtained using the IE method. Some of these blocks are carefully inspected after cutting to judge the true inner state. In practice, this can be done only in a small number of blocks, so we have a semi-supervised scenario.

The method proposed in this paper assumes knowledge of the number of classes. The multivariate probability density of the feature vectors corresponding to a particular class is considered to be a mixture of Gaussians (MoG). An MoG, also referred to as a Gaussian mixture model (GMM), assumes that all of the feature vectors for a given class are generated from a weighted sum of a finite number of Gaussian distributions with unknown parameters (mean and covariance). Hence, every feature vector is generated by one of the mixture components of a given class.

A given feature vector can be originated, in principle, by any component of any of the classes. However, labeled features are known to be generated by one of the components corresponding to the labeled class, although the specific component inside the class is unknown. This knowledge can be incorporated into the estimation of the whole model parameters, thus improving the performance of a Bayes classifier.

In Section 2, the new semi-supervised method is presented. Then, in Section 3, the dataset and the IE experimental setup are described. In Section 4, we present exhaustive results considering different target classes and levels of supervision, followed by the conclusions.

## 2. Method

There are two basic paradigms in order to define a classifier: a discriminative approach, where the goal is to directly assign the observations to the correct class, obtaining a rule that tries to minimize the errors, and a generative model approach, where we try to learn how the observations are generated, and after that, we assign the observation to the model with the highest probability. We will follow this second approach using the Bayesian classifier.

The Bayesian classifier calculates the posterior distribution *p*(*k*/x) for every class *k*, *k* = 1 … *K* and labels the observation x = [*x*_1_, … , *x_d_*]*^T^* with the class that has the largest probability. Applying Bayes' rule, we obtain:

(1) $$p(k/\text{x})=\frac{p(\text{x}/k)p(k)}{\overset{K}{\underset{k=1}{\text{∑}}}p(\text{x}/k)p(k)}$$

where *p*(x/*k*) is the conditional probability density of an observation vector x for class *k, k* = 1, 2, …, *K* and *p*(*k*) is the corresponding prior distribution for every class with $\overset{K}{\underset{k=1}{\text{∑}}}p(k)=1$.

The conditional distributions are modeled by an MoG for every class. An MoG is a weighted sum of Gaussians with mean μ*_i_* and covariance matrix Σ*_i_*.

(2) $$\begin{array}{l} p(\text{x}/k)=\overset{I_{k}}{\underset{i_{k}=1}{\text{∑}}}\alpha_{i_{k}}N_{i_{k}}(x;\mu_{i_{k}},\sum_{i_{k}})\\ N_{i_{k}}(\text{x};\mu_{i_{k}},\sum_{i_{k}})={\left(2\pi \right)}^{-d/2}{\left|\sum_{i_{k}}\right|}^{-1/2}e^{-\frac{1}{2}{\left(x-\mu_{i_{k}}\right)}^{T}\sum_{i_{k}}^{-1}\left(\text{x}-\mu_{i_{k}}\right)} \end{array}$$

where *I_k_* is the number of Gaussians used in the MoG that models the conditional distribution of class *k*.

For every class, each Gaussian contributes to the mixture model in the proportion or mixing coefficient *α*_*i_k_*_, with *α*_*i_k_*_ ≥ 0 and $\overset{I_{k}}{\underset{i_{k}=1}{\text{∑}}}\alpha_{i_{k}}=1$. These weights can also be interpreted as priors, indicating the prior probability of the data coming from the corresponding Gaussian of the mixture.

When an observation x is available, we can apply Bayes' theorem to calculate the posterior probability in Equation (1), *i.e.*, the probability that the observation comes from each class, and classify accordingly.

However, we need to estimate the previous model parameters. They include the prior probabilities of every class and the parameters of the different mixture models. In order to do this, we define the log-likelihood function *L*(**X**) of *N* observations **X** = [x_1_, …, x*_N_*]:

(3) $$L(\text{X};\Psi )=\log p(\text{X};\Psi )=\sum_{n=1}^{N}\log p({\text{x}}_{n};\Psi )=\sum_{n=1}^{N}\log\sum_{k=1}^{K}p({\text{x}}_{n}/k)p(k)$$

with the set of parameters to be estimated Ψ = {Ψ_1_,…, Ψ*_k_*}, where Ψ*_k_* = {*p*(*k*), *α*_*i_k_*_, μ_*i_k_*_, Σ_*i_k_*_}.

Using Equation (2) in Equation (3), we obtain:

(4) $$L(\text{X};\Psi )=\sum_{n=1}^{N}\log\sum_{k=1}^{K}p(k)\sum_{i_{k}=1}^{I_{k}}\alpha_{i_{k}}N_{i_{k}}({\text{x}}_{n};\mu_{i_{k}},\sum_{i_{k}})$$

The maximum likelihood estimator calculates the parameters that maximize Equation (4), *i.e.*, $\Psi_{\text{MLE}}=\underset{\Psi }{\arg\max}L(\text{X};\Psi )$. Since this equation involves the log of a sum, it is not easy to find the maximum, and an expectation-maximization (EM) approach [19] is better suited.

As usual, we assume that the observations **X** are part of a complete dataset (**X**, **Z**), **Z** = [**z**_1_,…, **z***_N_*], where **z***_n_* is a random vector of dimension $I=\overset{K}{\underset{k=1}{\text{∑}}}I_{k}$. This vector is equal to zero, but one element, which is equal to one, the class and Gaussian component that is responsible for the observation x*_n_*. *i.e.*,:

(5) $$z_{n}=[\underset{I_{1}}{\underset{︸}{0,\ldots 0}},\ldots ,\underset{I_{k}}{\underset{︸}{0,\ldots ,1,\ldots 0}},\ldots ,\underset{I_{K}}{\underset{︸}{0,\ldots 0}}]$$

with the one corresponding to the *m*-th element, *i.e.*, the *k*-th class and *i_k_*-th component in the mixture model of the *k*-th class conditional distribution.

The corresponding complete data log-likelihood reads:

(6) $$L_{c}(X,Z;\Psi )=\log p(X,Z;\Psi )=\sum_{n=1}^{N}\log p(x_{n},z_{n};\Psi )$$

Using Equation (5)*L_c_*(**X**, **Z**; Ψ) can be expressed as:

(7) $$\begin{array}{l} L_{c}(X,Z;\Psi )=\log\overset{N}{\underset{n=1}{\text{∏}}}\overset{I}{\underset{m=1}{\text{∏}}}{\left(p(x_{n}/z_{nm}=1)p(z_{nm}=1)\right)}^{z_{nm}}=\\ \overset{N}{\underset{n=1}{\text{∑}}}\overset{I}{\underset{m=1}{\text{∑}}}z_{nm}(\log p(x_{n}/z_{nm}=1)+\log p(z_{nm}=1)) \end{array}$$

Following the EM procedure, we first take the expectation of the complete log-likelihood. This expectation is obtained assuming that the model parameters are fixed Ψ = Ψ*^j^*, *i.e.*, the class probabilities, proportions, means and covariance matrices are fixed. If the expectation is taken with respect to the posterior distribution of the unobserved data, it is guaranteed that maximizing the complete log-likelihood, we are also maximizing the incomplete log-likelihood function, which is the real problem. This is the expectation step in the EM algorithm and can be summarized as:

(8) $$Q(\Psi ;\Psi^{j})=E[\log p(X,Z;\Psi )/X,\Psi^{j}]$$

Substituting:

(9) $$Q(\Psi ;\Psi^{j})=\sum_{n=1}^{N}\sum_{m=1}^{I}E[z_{nm}](\log(p(x_{n}/z_{nm}=1)+\log\delta_{m})$$

where *δ_m_* = *p*(*z_nm_* = 1), with the constraint $\overset{I}{\underset{m=1}{\text{∑}}}\delta_{m}=1$.

The expectation step calculates the expected value of the responsibilities using the present values of the parameters:

(10) $$\begin{array}{c} E[z_{nm}/x_{n},\Psi^{j}]=1\;\cdot \;p(z_{nm}=1/x_{n},\Psi^{j})+0\;\cdot \;p(z_{nm}=0/x_{n},\Psi^{j})=\\ =p(z_{nm}=1/x_{n},\Psi^{j}) \end{array}$$

The posterior probabilities *p*(*z_nm_* = 1/x*_n_*, Ψ*^j^*), *i.e.*, the responsibility that the element *m*-th takes for generating the *n*-th observation given the current model parameters, are obtained using Bayes' theorem:

(11) $$p(z_{nm}=1/x_{n},\Psi^{j})=\frac{p(x_{n}/z_{nm}=1)p(z_{nm}=1)}{\overset{I}{\underset{m=1}{\text{∑}}}p(x_{n}/z_{nm}=1)p(z_{nm}=1)}=\frac{p(x_{n}/z_{nm}=1)\delta_{m}}{\overset{I}{\underset{m=1}{\text{∑}}}p(x_{n}/z_{nm}=1)\delta_{m}}$$

Once the expectation is calculated, Equation (7) is no longer a random variable; we have just a log-likelihood function *Q*(Ψ; Ψ*^j^*) that can be maximized as usual with respect to the model parameters, obtaining a new estimate of them Ψ*^j^*^+1^; this is the maximization step of the algorithm:

(12) $$Q(\Psi ;\Psi^{j})=\sum_{n=1}^{N}\sum_{m=1}^{I}p(z_{nm}=1/x_{n},\Psi^{j})(\log(p(x_{n}/z_{nm}=1)+\log\delta_{m})$$

This two-step procedure is repeated iteratively until convergence, and it is guaranteed that in every iteration, the likelihood function is increased and, therefore, converges to a local maximum.

To obtain the new parameters, we have to maximize *Q*(Ψ; Ψ*^j^*). In order to maximize *Q*(Ψ; Ψ*^j^*), we take derivatives with respect to every parameter of the model and set them equal to zero. Note that the function *Q*(Ψ; Ψ*^j^*) can be decoupled in a sum of different terms, where each of them includes only one kind of parameter, *i.e.*, $\delta_{m}^{j+1}$, $\mu_{m}^{j+1}$, $\sum_{m}^{j+1}$. Remember that index *m* is related to the corresponding *i_k_* in Equation (2) depending on the number of classes and Gaussians per class model.

Once the objective function *Q*(Ψ; Ψ*^j^*) is decomposed, we can obtain the new mean, variance and component weight in the same way as in the classic EM algorithm:

(13) $$\mu_{m}^{j+1}=\frac{\overset{N}{\underset{n=1}{\text{∑}}}p(z_{nm}=1/x_{n},\Psi^{j})x_{n}}{\overset{N}{\underset{n=1}{\text{∑}}}p(z_{nm}=1/x_{n},\Psi^{j})}$$

(14) $$\sum_{m}^{j+1}=\frac{\overset{N}{\underset{n=1}{\text{∑}}}p(z_{nm}=1/x_{n},\Psi^{j})(x_{n}-\mu_{m}^{j}){\left(x_{n}-\mu_{m}^{j}\right)}^{T}}{\overset{N}{\underset{n=1}{\text{∑}}}p(z_{nm}=1/x_{n},\Psi^{j})}$$

(15) $$\delta_{m}^{j+1}=\frac{1}{N}\overset{N}{\underset{n=1}{\text{∑}}}p(z_{nm}=1/x_{n},\Psi^{j})$$

We estimate the new probability for every class *p*(*k^j^*^+1^) integrating out the corresponding $\delta_{m}^{j+1}$ elements:

(16) $$p(k^{j+1})=\sum_{m=m_{0}+1}^{M}\delta_{m}^{j+1}$$

where the indexes of the summation are:

(17) $$\begin{array}{c} m_{0}=\overset{k-1}{\underset{j=1}{\text{∑}}}I_{j}\\ M=\overset{k}{\underset{j=1}{\text{∑}}}I_{j} \end{array}$$

The new weights for every Gaussian component are obtained normalizing the full responsibility by the corresponding class probability:

(18) $$\alpha_{m}^{j+1}=\frac{\delta_{m}^{j+1}}{p(k^{j+1})}$$

In the case that all classes have the same number of Gaussians, the notation simplifies to:

(19) $$p(k^{j+1})=\sum_{m=(k-1)R+1}^{kR}\begin{array}{cc} \delta_{m}^{j+1}, & k=1,\ldots ,K \end{array}$$

(20) $$\begin{array}{cc} \alpha_{m}^{j+1}=\frac{\delta_{m}^{j+1}}{\overset{\left\lceil m/R\right\rceil R}{\underset{n=\left\lfloor m/R\right\rfloor R+1}{\text{∑}}}\delta_{n}^{j+1}}, & m=1,\ldots ,KR \end{array}$$

where ⌊.⌋, ⌈.⌉ are the floor and ceiling operators, respectively.

Supervision is introduced implicitly in Equation (11). The posterior probability *p*(*z_nm_* = 1/x*_n_*, Ψ*^j^*) for samples with a known class *k* is easily computed, such as:

(21) $$p(z_{nm}=1/x_{n},\Psi^{j})=\frac{p(x_{n}/z_{nm}=1)\alpha_{m}}{\overset{I}{\underset{m=1}{\text{∑}}}p(x_{n}/z_{nm}=1)\alpha_{m}}$$

for the interval of indexes *m* corresponding to the components of the mixture model of the known class *k* with priors *α_m_* and *p*(*z_nm_* = 1/x*_n_*, Ψ*^j^*) = 0 for the rest of indexes *m*.

Since this posterior probability is used in the maximization step, it means that samples x*_n_* that belong to a known class *k* are used to update the parameters of that class only: mean, covariance and prior probabilities of the MoG for that class; see Equations (13)–(15). In other words, the rest of the classes do not take into account those samples in the updating step of their parameters, since in their sums, those terms are zero, *p*(*z_nm_* = 1/x*_n_*, Ψ*^j^*) = 0, for indexes *m* out of the interval corresponding to the known class *k*.

**Figure 1:**
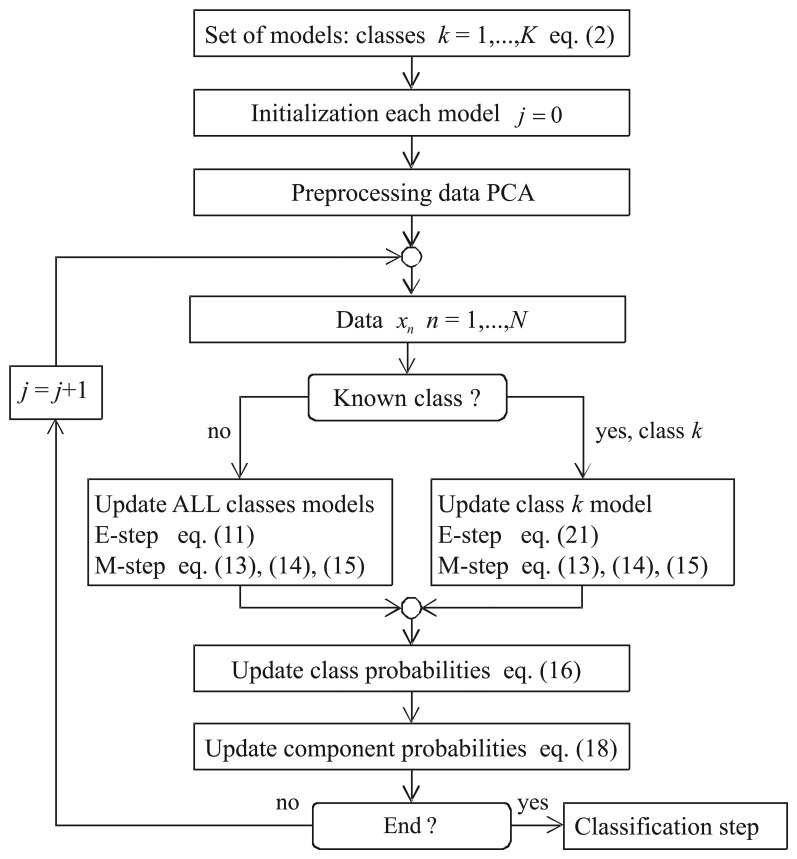
Flowchart of the algorithm.

The algorithm is summarized in Figure 1. First, you set the models (one MoG per class) and the parameters (how many Gaussians per class). Second, the mean and covariance matrices of each class are initialized. For this purpose, only the samples from a known class (supervised samples) are used in the initialization of the corresponding class parameters. Third, the data are preprocessed using PCA in order to reduce the dimensions of the feature vector. Fourth, the algorithm is run until convergence. The algorithm stops when the new and old parameters change less than a threshold value.

## 3. Data

**Figure 2:**
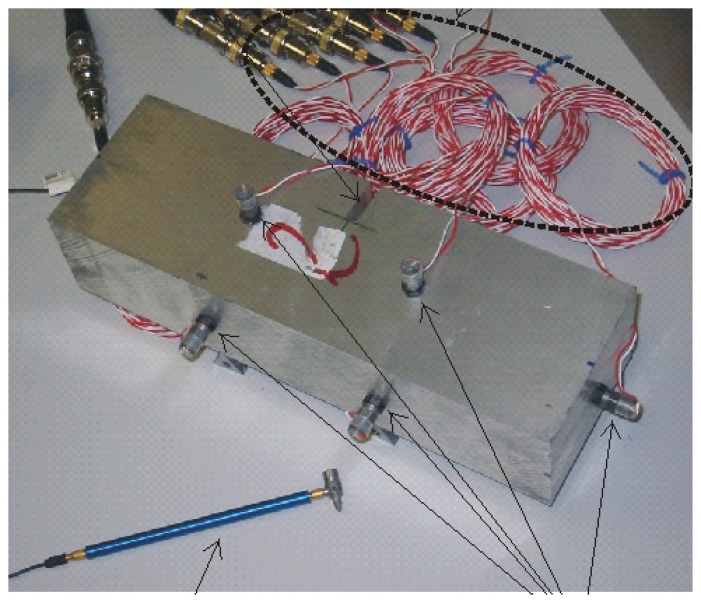
Example of the piece under study.

We apply the explained algorithm to real data obtained in the lab using materials made of aluminum alloy series 2000 of dimensions 7 × 5 × 22 cm (width, height and length, respectively). These dimensions were appropriate for lab experiments and may be considered reasonable scale replicas of real specimens used in different problems were the impact echo method has been applied (see, for example, [20–22] and the references therein). Moreover, these dimensions are appropriate for a dense excitation of resonant modes [23], thus leading to rich spectrum content. We show an example of a piece under study in Figure 2: arrows point to the hammer and the accelerometers, while the red and white cables connect the accelerometers to the acquisition equipment.

Up to three defects per piece were drilled in different locations of each piece. The defects passed through the pieces and consisted of holes in the shape of cylinders of 10 mm and cracks in the shape of parallelepipeds of 5 × 20 mm cross-sections. Some of the defects cross all of the pieces, e.g., a hole that passes totally through the other face of the material, and some others do not, stopping at some point in the interior of the piece. The material was excited by an impact, and its response was measured by the accelerometers (sensors).

**Figure 3:**
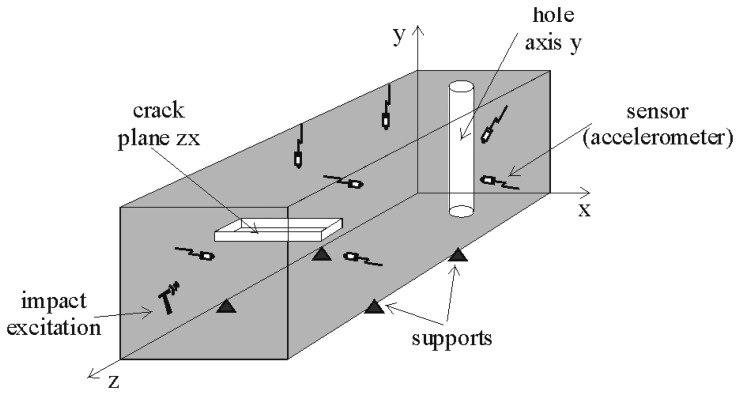
Setup experiment for a piece with a hole and a crack.

As an example, in Figure 3, we show the setup for a piece with a hole and a crack defect. We use seven sensors located on different surfaces of the parallelepiped in order to capture the information coming from different directions and distances from the impact and defects. In the example provided in the figure, the hammer impacts on the front face. There is a hole in the Y axis far from the impact surface and one crack in the XZ plane near the impact plane.

With respect to the equipment, we used an impact hammer 084A14 PCB, eight accelerometers (a1–a8 in the figure) 353B17 PCB, an ICPsignal conditioner F482A18 and a data acquisition module 6067E. The acquisition parameters were: sampling frequency = 100,000 kHz and observation time = 50 ms. We use as input data the spectrum of the recorded signals coming from the sensors, normalized using the maximum of the impact signal amplitude. The total number of experiments included 1881 executions of the IE test from 76 specimens.

The 76 pieces can be grouped into different classes attending to several criteria: the status of the piece, the kind of defect, the orientation of the defect and the length of the defect. Depending on the criteria we use, we can state different classification problems with an increasing number of classes. The first problem has four classes, since the pieces are divided into four groups: non-defective (also called homogeneous), one hole defect, one crack defect and multiple defects.

If we split the one defect pieces into subclasses attending to the orientation of the defect, we have a second problem with eight classes: homogeneous, one hole in the X axis, one hole in the Y axis, one hole in the Z axis, one crack in the XY plane, one crack in the YZ plane, one crack in the XZ plane and multiple defects.

The most challenging case is when we also use the length of the defect: if it goes through the piece or just up to some point in between, we call these subclasses passing through and not passing through, respectively. Thus, in the most complex case, we have fourteen classes.

Due to technical reasons and for simplicity in the making of the specimens in the lab, we have no Z direction hole pieces, nor obviously passing through and not passing through Z direction hole samples. Therefore, the three classification problems that we address have four, seven and twelve classes, respectively.

All of the information about the specimens and data collection is summarized in Table 1.

One important issue in any classification procedure is to take care of the dimensions of the data during the preprocessing of the data. Since we have to estimate some parameters, it is important to be sure that the estimates are accurate enough to prevent possible overfitting. In our case, this means that we have to reduce the dimensions of the feature vector: the spectrum of the signals captured by the accelerometers. To this end, we preprocess the data applying PCA, as explained in [24]. The number of PCA components that we keep is given by a threshold on the fraction of variance captured by those components.

Another important factor is the number of samples that are available, *i.e.*, the total amount of data available during the learning process. On the one hand, the pieces made in the lab were submitted to different impacts in order to increase the number of samples and to introduce some randomness and noise in the recording process, since the impact is slightly different in every experiment. On the other hand, since the mechanical process of making pieces with specific defects is difficult, we use resampling techniques to increase the size of the sample when necessary [25]. This consists of generating new realizations, called replicas, by adding to the real recorded value a small amount of white Gaussian noise with a small standard deviation. This helps to improve the learning process, and by using cross-validation methods, we can assure that no overfitting problems arise.

## 4. Results

We applied the classifier to the dataset explained in the previous section. We split the samples into two groups: a training set containing 80% of the data and a testing set with the rest of the samples. In order to cross-validate the results, we ran the algorithm 40 times for every experiment with different training-test data, and we show the calculated mean values.

### 4.1. Measures

To quantify the results, we use a confusion matrix [26]. We define the confusion matrix as a matrix where every row represents the estimated class (the result of our algorithm) and every column the true class (the solution); note that the transpose definition (exchanging rows and columns) could have also been used. With *K* classes, the confusion matrix is *K* × *K*, where the diagonal entries correspond to the correct classifications. The off-diagonal values in every row tell us how the wrong classifications for that estimated class (false positives) are distributed among the true classes and the off-diagonal values in every column how many specimens from a given class are wrongly assigned to each of the other classes (false negatives). This matrix contains all of the information about the performance of the algorithm for our dataset, but it can be tedious to analyze the results and obtain simple conclusions from the confusion matrix. Therefore, we will use also other measures obtained from the confusion matrix to clarify the results.

Since we are considering the same cost for every wrong classification, we are not interested at this point in comparisons between particular classes. Therefore, we can obtain *C_i_*, *i* = 1 … *K* confusion matrices 2 × 2, where for each *C_i_* matrix, the *i*-th class is the positive one and the aggregate of the rest of them is the negative one (errors). These matrices are easily obtained by simply summing up the corresponding values of the whole confusion matrix. The *C_i_* matrices allow us to obtain the precision *p_i_* and recall *r_i_* values for every class *i* = 1 … *K*:

(22) $$p_{i}=\frac{TP_{i}}{TP_{i}+FP_{i}}$$

(23) $$r_{i}=\frac{TP_{i}}{P_{i}}$$

where *TP_i_* are the true positives (when the estimated and true classes are the same *C_i_*), *FP_i_* are the false positives (when we assign the piece to the positive class *C_i_* erroneously, no matter which is the true class) and *P_i_* is the total number of specimens of the *i*-th class. In other words, precision indicates the ability of the algorithm to distinguish between true and false positives, *i.e.*, how many of the pieces assigned to class iare correct; a large *p_i_* value indicates that most of the pieces that are assigned to class *i* actually belong to that class. Recall, also called the true positive rate or sensitivity, indicates the ability to detect the positive cases, *i.e.*, how many of the pieces from the *i*-th class are detected; a large *r_i_* value indicates that most of the pieces from that class are identified. We can even reduce these two indices to just one, combining them properly; e.g., the *F* measure, which is defined for every class as the harmonic average of precision and recall values:

(24) $$F_{i}=\frac{2}{\frac{1}{p_{i}}+\frac{1}{r_{i}}}$$

The values of precision, recall and the F measure are between zero and one, with larger values indicating better performance. In order to make the comparison easier between results, we will use percentage values, *i.e.*, in the interval 0–100. It is important to remark that these values must be used with care, especially in cases where the class distributions change or are skewed and when the cost functions are not 0–1 loss functions (no cost to correct classification and the same cost for all errors). In our experiments, we do not have these problems, since we use the same equiprobable class distributions during the training and testing stages, and as we mentioned, we will consider the same cost for any wrong classification.

### 4.2. General Results

In this subsection, we analyze the general behavior of the algorithm no matter which of the three problems we are solving. We are not interested in how the algorithm performs for any particular class, but we want to extract general conclusions about the performance of the algorithm and how it is influenced by different variables, such as the model parameters or the data size.

**Figure 4:**
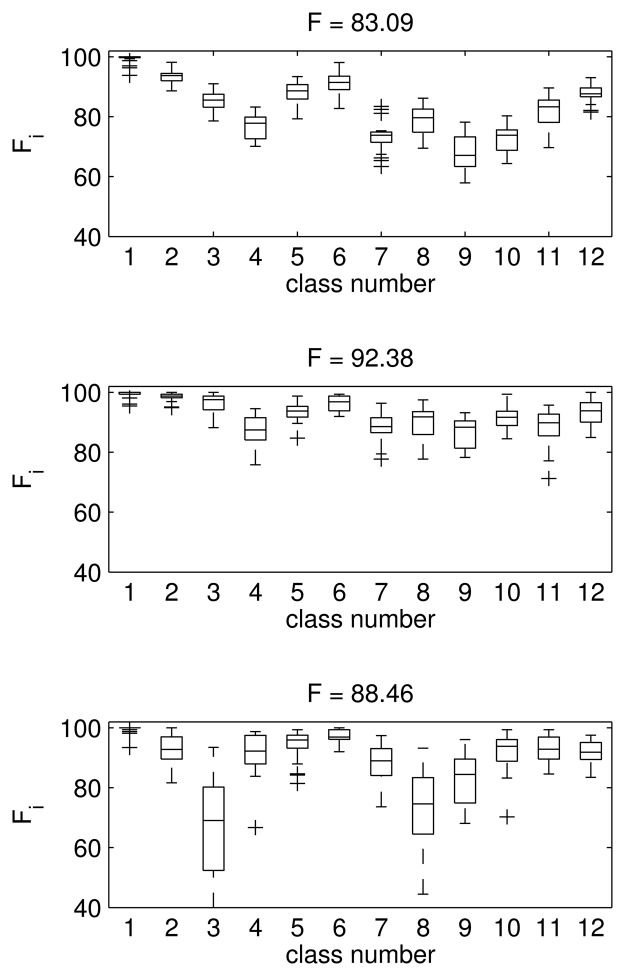
Dimension reduction of the feature vector after PCA. Box and whiskers plot of the F measure for each of the 12 classes. (**Top**) 3 × 1 feature vector; (**Middle**) 7 × 1 feature vector; (**Bottom**) 16 × 1 feature vector. Above each plot is the overall mean F measure.

The first thing we have to establish is the dimension of the feature space, *i.e.*, the number of principal components that we are going to keep after PCA. We will use the 12-class problem to determine the feature space dimension, since it is the most complicated case. To analyze the influence of the dimension of the feature vector, we run the algorithm for different dimensions. We obtain the confusion matrix and then calculate the F value. In Figure 4, we show the box and whiskers plot of the F value for all of the classes and the overall F mean value (40 runs for each one) when the feature vector is a 3 × 1 vector (top), a 7 × 1 vector (middle) and a 16 × 1 vector (bottom). These values correspond to keeping 25%, 50% and 75% of the total amount of the variance when applying PCA. The overall F mean values are: 83.09, 92.38 and 88.46, respectively.

As we can see in Figure 4, the best results are obtained in the middle case, when the data are projected to a seven-dimensional PCA space. When we increase the dimensions from three to seven, the results improve dramatically: the mean F value goes from 83.09 to 92.38. However, if we increase the number of descriptors to 16, then the results for the test data are worse, as shown in the bottom plot (F reduces to 88.46). This fact is especially clear for Classes 3, 8 and 9, with a large variance and a poor mean classification performance. These results correspond to the case of nine Gaussians per class and a 0.3 supervision ratio. However, we obtain the same conclusion for a wide range of numbers of Gaussians (*I_k_* in Equation (2)) and supervision values (percentage of known labels used in the training stage). Therefore, we will use the first seven principal components of the PCA transformation of the spectrum as the feature vector.

Once the preprocessing is done, we address the influence of the model complexity on the performance of the classifier, *i.e.*, how to choose the number of Gaussians per class. We seek a number of Gaussians large enough to capture the distribution of the class, but not too high to avoid overfitting. We assume that the complexity of every class is similar, so we will use the same number of Gaussians per class. In such a way, we will not bias the results in favor of any class. With respect to the initialization of the parameters of each Gaussian component for every class (the mean, covariance matrix and proportions of every component), we use the set of supervised samples. The mean of every component corresponds to a random known value of the corresponding class, and the initial covariance matrix is the same for all of the components in the same class: it is obtained as the sample covariance from the known samples, and the components are equiprobable. The initial class priors are calculated obtaining the proportion of every class in the total number of samples with a known class. We used a similar number of samples from every class in all of the problems, so we do not have to worry about the influence of the prior in the evaluation of the results.

**Figure 5:**
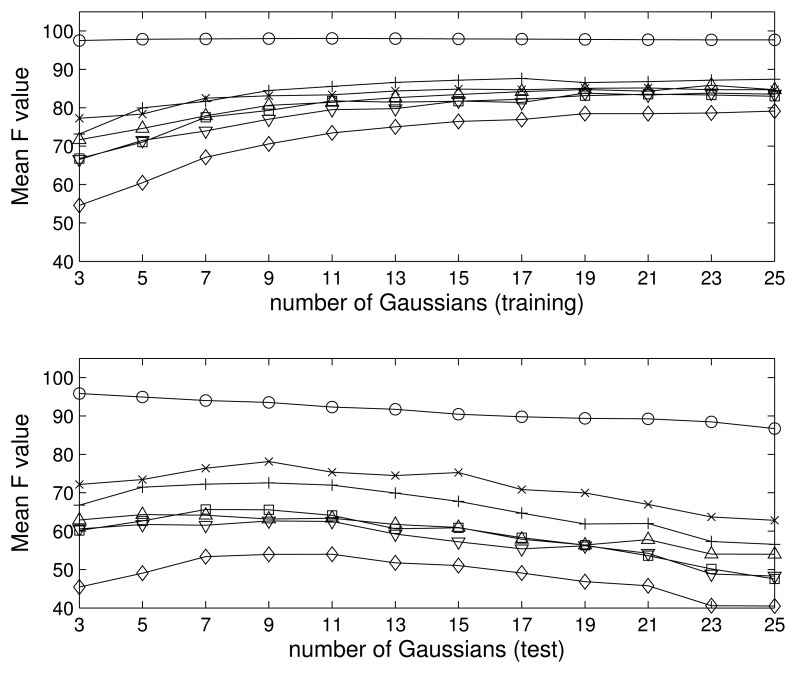
Model selection: number of Gaussians per class for the seven classes problem. (**Top**) Mean F value of each class for the training dataset *vs*. the number of Gaussians per class; (**Bottom**) the same for the testing dataset.

We trained and tested the model for an interval of Gaussians, from just three up to 25, in steps of two, *i.e.*, *I_k_* = 3, 5, … , 25. In Figure 5, we show the F value obtained for the problem of seven classes during the training (top) and test (bottom) phases. The training results improve as the complexity of the model does, since we can always introduce new Gaussians in order to fit the data better. However, it is clear that the algorithm suffers from overfitting for a number of Gaussians approximately greater than nine, since the test performance is reduced while the training improves; *i.e.*, for a large number of Gaussians, the model is so complex, that it can fit to the training data, but is not able to generalize to new data. To avoid this problem, we restrict our model to a maximum of nine Gaussians per class.

The supervision ratio depends on the available data. It is expected that if the number of samples with a known class increases, the performance of the algorithm should be better. Note that in the extreme case where we know which class any training sample belongs to, our algorithm reduces to the estimation of every class mixture model through the standard EM algorithm. Once the different distributions are obtained, it would be easy to assign the new data to the class with higher probability. On the other hand, for the unsupervised case, every sample will contribute to every mixture model, so it could become an unsolvable problem if modes of different class distributions overlap, because it would be impossible to assign it to one model or another. The algorithm could find the correct Gaussian components in the overall observation distribution, but it could assign the components to the wrong class. If this is the case, the only solution is to introduce some level of supervision in order to guarantee that the components are assigned properly to the corresponding class. In addition, as explained previously, we cannot afford to have a very large number of samples of every class, since it would be very expensive and time consuming. That is to say, it would be a nice feature of the classifier to learn from a reduced number of samples. Therefore, we need to test the influence of the supervision ratio and sample size in the performance of the algorithm in order to quantify this effect.

**Figure 6:**
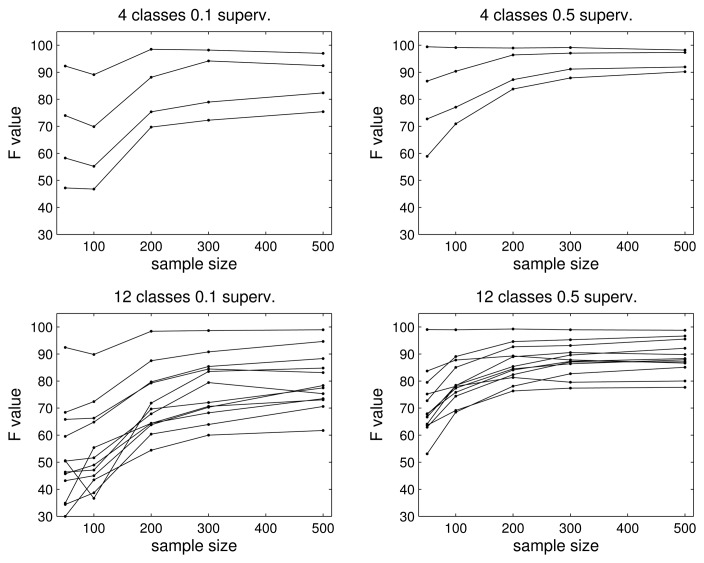
Influence of data size and supervision. (**Top**) Four-class problem; (top-left) mean F value for the four classes *vs*. the number of samples per class for a 10% supervision ratio; (top-right) results with 50% of supervision; (**Bottom**) 12-class problem; (bottom-left) mean F value for the 12 classes for a 10% supervision ratio; (bottom-right) results with 50% supervision.

To analyze the influence of the data size and supervision, we obtain the results for different sets of samples per class, 50, 100, 200, 300 and 500, and two different supervision rates, 0.1 and 0.5. In Figure 6, we show the results for the four- and 12-class problems. Again, we use the F value in the figures to simplify the analysis.

As we can see, the algorithm needs a minimum amount of data of around 200 samples per class to obtain good results in both problems, *i.e.*, the sample size matters: if it is very low, e.g., 50 or 100, the classification rate can be poor (as low as an F value of 0.5 or 0.3 for the four- and 12-class problems, respectively). However, after some number of samples (300 in our experiments), increasing the number of samples does not improve the results significantly. The only way to increase the classification rate is by introducing more supervision. In other words, as was expected, the supervision factor helps to obtain better F values, since more samples are used exclusively for training the mixture model of the corresponding class. Therefore, in a real application, the first goal is to achieve a minimum number of samples for every class so that the estimates can achieve a minimum quality. After that, if we can obtain more samples with known labels, we know that the performance will improve, since the problem is no longer about the amount of data, but the quality of the data.

**Figure 7:**
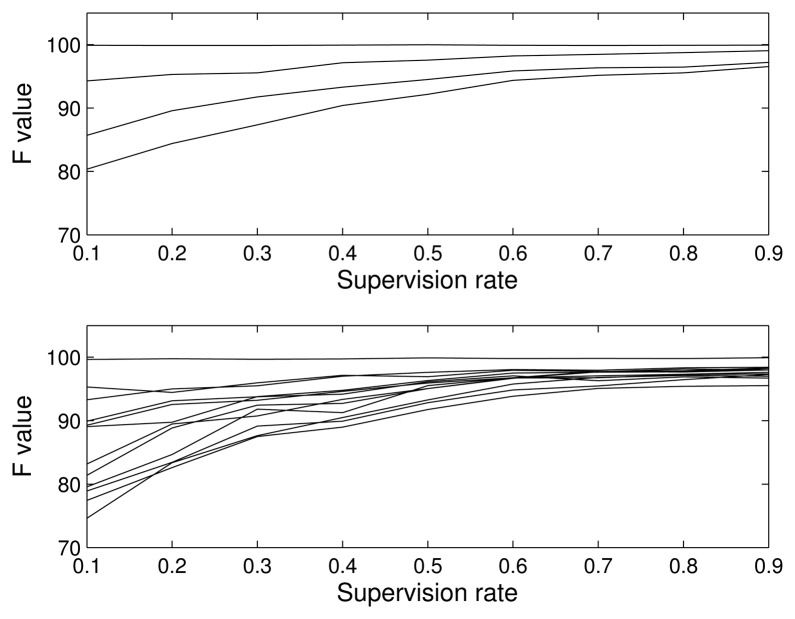
Influence of supervision. F value vs. percentage of supervision. (**Top**) Four-class problem; (**Bottom**) 12-class problem.

To clearly see the effect of the amount of supervision on performance, we show in Figure 7 the F value for different supervision rates: 0.1 (almost unsupervised), 0.3, 0.5, 0.7 and 0.9 (almost supervised). As was expected, the supervision allows one to model the distributions better, so the classification accuracy improves. Note that, even in the case of almost completely supervised classification, the classification is not perfect, since the classes are not separable, but the values are very close to a perfect classification, showing the good behavior of our algorithm.

### 4.3. Results Depending on the Kind of Defect

Until now, we have studied the general performance of the algorithm, obtaining conclusions about the preprocessing of the feature vector (PCA dimensions reduction), the complexity of the model (how many Gaussians) and how the data size and supervision affects the results of the algorithm. Now, we will proceed to the analysis from the point of view of a single class.

In all previous figures, regardless if the 4-, 7- or 12-class problem was used, there was almost always a class that was perfectly classified even in inappropriate conditions (a feature vector with very few dimensions, bad class probability models with a small number of Gaussians and bad estimates of the model with few data and no supervision). This class was always the homogeneous material. It is quite logical to assume that a material that has no defect has a very different spectrum from defective materials. In a real implementation, we have no idea about the prior probabilities of the classes. We do not know if there are more defective than non-defective pieces or *vice versa*. Therefore, we have to remark again on the importance of using classification measures that are not sensitive to the prior probabilities of the classes; e.g., if we always decide that the piece is homogeneous and the prior of the non-defective material class is 0.95, we will obtain a 0.95 true positive rate, which can be misunderstood as the good performance of the algorithm if we only consider this measure.

The same could be said about the cost function. In a real implementation, attending to many other variables, such as the revenues, the owner of the system can change the decision making threshold in order to obtain the true positive/false alarm rate that is better for him; e.g., if many pieces are being classified as defective, he can change the decision rule that changes the cost function or, equivalently, assigning the piece to the defective class only when the posterior probability is higher than a given value instead of assigning it to the class with higher posterior probability.

**Figure 8:**
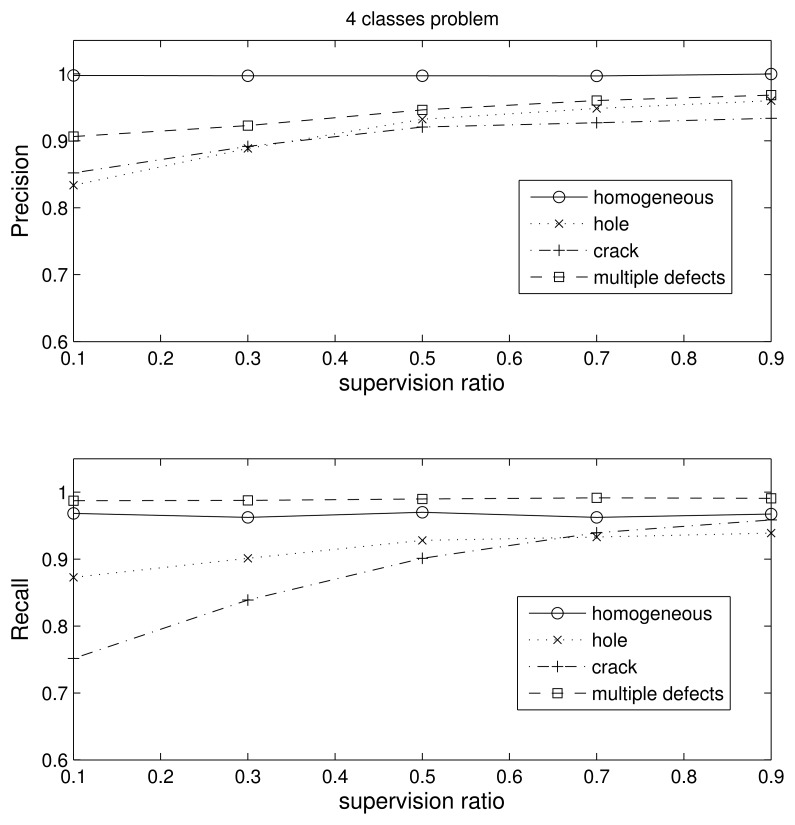
Precision and recall for the four-class problem vs. the supervision ratio.

In this section, we use nine Gaussians per class and 500 samples per class during training. The four-class problem essentially tests the ability of the algorithm to discriminate between defective and non-defective pieces. In Figure 8, we obtain the precision and recall values for each of the four classes (homogeneous, hole defect, crack defect and multiple defects) with respect to the supervision ratio, and in Table 2, we show the confusion matrix for the case of 0.1 supervision (the worst case).

As we can see in the figure, the results are very good. The precision of the homogeneous class is nearly 100% for any supervision value. In the case of low supervision, the precision is a little bit lower than 90% for the pieces with multiple defects and almost equal to 85% for the one defect blocks. However, the recall is almost perfect for the homogeneous and multiple defect classes in all supervision scenarios, *i.e.*, almost no piece in these classes is missed, regardless if they are included during the learning process. The lowest precision value of 75% is for the pieces with a crack and 0.1 supervision. Note that low supervision is the most realistic case for industrial applications, since most defects are not observable and we want to retain the largest amount of pieces. Looking at Table 2, we see that most of the errors occur when one crack and hole classes are confused; *i.e.*, the cost would be drastically reduced if we consider that the kind of defect does not matter and we merge the hole and crack class into just one class.

**Figure 9:**
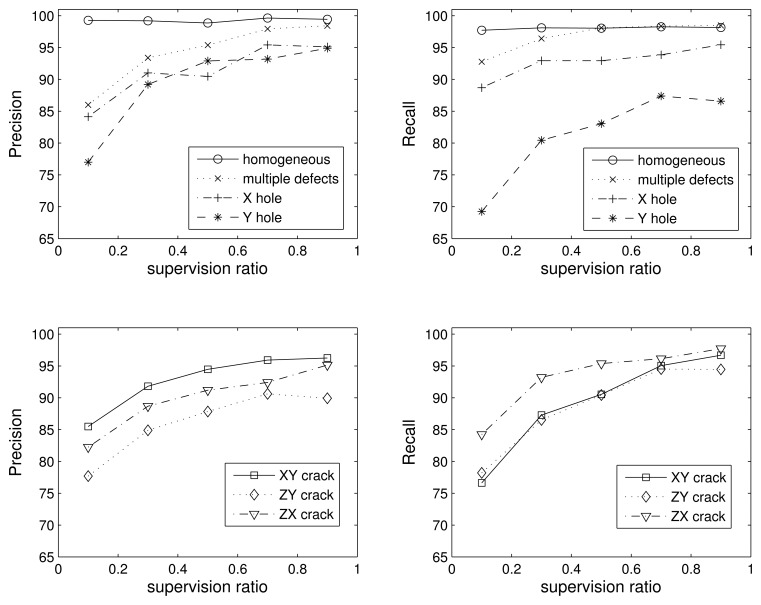
Precision and recall for the seven-class problem vs. supervision ratio.

In the seven-class problem, we split the hole and crack classes into subclasses, taking into account the direction of the defect: homogeneous, X hole, Y hole, XY crack, ZY crack, ZX crack and multiple defects. In Figure 9, we show the precision and recall values for the seven classes. For the sake of clarity, we split the results into different subplots.

Again, the homogeneous class is the best one, and increasing the supervision improves the performance for all classes. If we analyze the errors between classes, we find that most of the mistakes are between the Y hole and ZY crack classes. The values in the confusion matrix are shown in Table 3 only for the case of 0.5 supervision. However, the same occurs for the rest of the supervision values. A detailed understanding of this aspect will require an in-depth analysis from the perspective of wave propagation, which is outside of the scope of this work. However, we may conjecture that due to the orientation of the impact and the similar width of both the ZY crack and the Y hole in the X direction (see Figure 3), the respective cross-sections “seen” by the impact point are similar, thus facilitating the confusion of these two kinds of defects.

**Figure 10:**
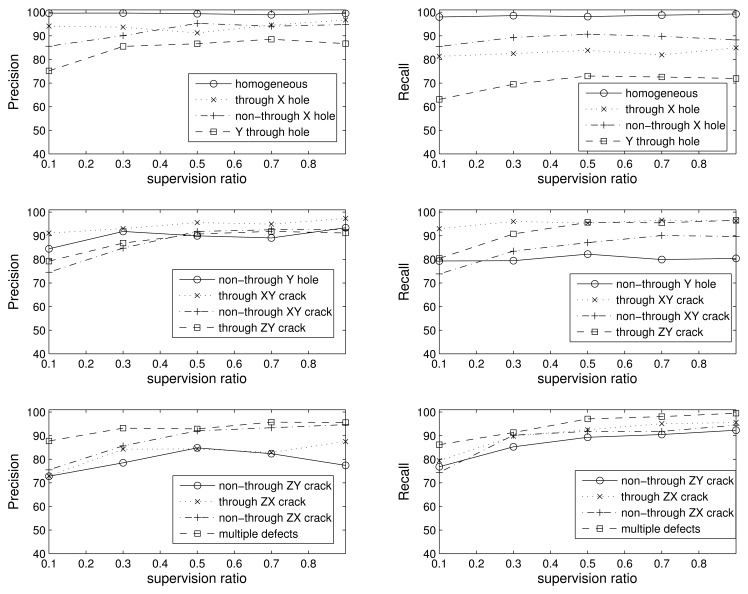
Precision and recall for the 12-classes problem vs. supervision ratio.

In the 12-class problem, we introduce another distinction between defects (passing through or not passing through): homogeneous, through X hole, non-through X hole, through Y hole, non-through Y hole, through XY crack, non-through XY crack, through ZY crack, non-through ZY crack, through ZX crack, non-through ZX crack and multiple defects. In Figure 10, we show the precision and recall values for each of the twelve classes with respect to the supervision ratio. For the sake of clarity, we split the results into different figures, each one corresponding to four out of the 12 classes. Since the problem is much more complex, the results are good, but worse than in the previous scenarios. The detailed analysis of the confusion matrix (not shown for the sake of readability) reveals the same conclusion as in the seven-class problem: most mistakes are between holes and cracks that are aligned in the direction of the impact hammer.

### 4.4. ROC Curves

Since the algorithm assigns *a posteriori* probability for every sample, we can use this probability value as a score to obtain an ROC curve for every class. ROC curves have two important characteristics. First, they are insensitive to class probabilities. Second, they provide information about detection rate *vs*. false alarm rate, so a user can choose the working point of the detector based on his preferences. Therefore, by analyzing the ROC graph, the user can choose the operating point (the pair true positive-false positive rates) according to his interest, e.g., a conservative detection system where we want to avoid the false positives, or just the point where the system obtains the best performance. According to this policy, we only have to adapt the threshold (score or probability) to the corresponding value. This is an important advantage of our Bayesian classifier with respect to other classification algorithms that only provide a binary decision, so it is difficult to evaluate the results beyond a misclassification point of view. Since we are working with up to twelve classes, it is important to know if the posterior distributions are similar or not, as this can give us an idea about the robustness of the classifier when the samples, priors or cost function change.

**Figure 11:**
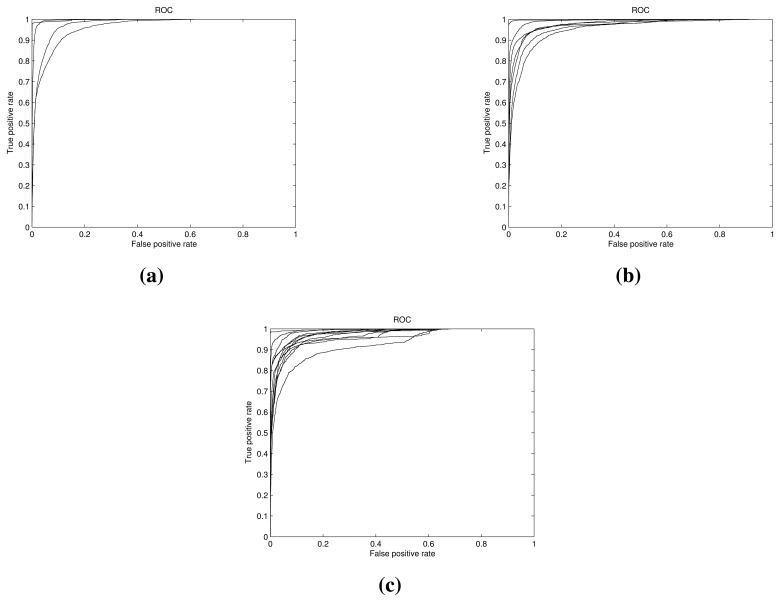
ROC curves. Detection rate vs. false alarm rate. (**a**) Four classes; (**b**) seven classes; (**c**) 12 classes.

In Figure 11, we show the ROC curves for the 4, 7 and 12 classes for the 0.1 supervision case. As we can see, the graphs are clearly over the diagonal line in all cases, exhibiting the good performance of the algorithm. With this information, the user can select the operating point without having to learn or adapt any parameter in the system; just changing the detection threshold results in the false alarm rate that he wants.

## 5. Discussion and Conclusions

The statement of the problem from a statistical point of view permits the use of a generative model that is helpful in the understanding of the problem. Since we obtain the posterior probability for every class, we can use it for simple classification or for a more sophisticated analysis based on ROC curves.

In an industrial application, such as in the marble industry, flexibility is a key factor, since the user needs an adaptable tool where the classifier can be modified easily by considering economic reasons. However, this flexibility is more helpful if the user can learn from it. In other words, we need to quantify the change in the results not only in terms of classification-misclassification rates, as typical discriminative classifiers do. This is an advantage of our algorithm, since the user can analyze the posterior probabilities and learn from them in order to define the classification rule that is appropriate for them.

We have used the MoG model to approximate the conditional distribution of the data given a class. We have explained how to estimate the number of Gaussians and the rest of the parameters in order to obtain good performance while avoiding overfitting. Of course, the final results will depend on the separability of the classes.

A very good characteristic of the proposed algorithm is that it does not require a large amount of samples to estimate the parameters properly. In addition, we have shown how the quantity and quality of the data, *i.e.*, the supervision ratio, complement each other. This means that in a real application, we do not require the destruction of many pieces to obtain enough data, and, therefore, the implementation is affordable.

The results show that the homogeneous class is easily separable from the rest of the classes. An interesting conclusion is that homogeneous and defective specimens are rarely misclassified. When the number of classes increases, *i.e.*, when the defective pieces are subdivided into different subclasses, the problem is not as easy. This is especially true for the specimens that have similar defects, e.g., a hole and a crack with the same orientation. In this case, the only way to improve the performance is to better estimate the model parameters by increasing the supervision ratio. The price to pay is the economic cost, as we need to destroy more specimens to determine to which class they belong.

In order to help those who want to replicate our experiments, we give some advice. First, run multiple impact-echo experiments and discard the first ones. Second, the size of the test specimen plays an important role in the signals. It is necessary to perform the impact-echo test at several points on the surface to identify possible geometrical effects. In summary, it is advisable to test first the variables that can affect the results and to be sure that, in case you cannot control them, at least you can reduce their effects by running the experiments under different conditions, so that the effects are averaged and the signals are not biased.

## Figures and Tables

**Table 1:** Dataset: number of pieces and experiments per class.

**Type of Defect**	**Number of Pieces**	**Number of Experiments**	**Size of Defect (mm)**
Homogeneous	6	200	-
Passing through a hole in the X axis	4	85	∅ =10, X:70
Half-passing through a hole in the X axis	4	89	∅ =10, X:35
Passing through a hole in the Y axis	4	84	∅ =10, X:50
Half-passing through a hole in the Y axis	4	83	∅ =10, X:25
Passing through a crack in the XY plane	8	170	X = 20, Y = 50, Z = 5
Half-passing through a crack in the XY plane	8	160	X = 20, Y = 25, Z = 5
Passing through a crack in the ZY plane	8	187	X = 5, Y = 50, Z = 20
Half-passing through a crack in the ZY plane	8	160	X = 5, Y = 25, Z = 20
Passing through a crack in the ZX plane	8	185	X = 70, Y = 5, Z= 20
Half-passing through a crack in the ZX plane	8	182	X = 35, Y = 5, Z = 20
Multiple defects	6	296	Combinations of cracks and holes

Total	76	1881	

**Table 2:** Confusion matrix for the 4-class problem (supervision 0.1).

	**Homogeneous**	**Hole**	**Crack**	**Multiple**
homogeneous	99.74	0.03	0.13	0.10
hole	0.28	83.05	15.99	0.68
crack	0.85	13.81	84.82	0.52
multiple	1.92	0.42	7.23	90.43

**Table 3:** Confusion matrix for the 7-class problem (supervision 0.5).

	**Homog.**	**X Hole**	**Y Hole**	**XY Crack**	**ZY Crack**	**ZX Crack**	**Multiple**
homog.	98.74	0.00	0.44	0.05	0.16	0.60	0.00
X hole	1.05	89.95	5.58	0.42	1.68	1.21	0.11
Y hole	0.06	1.76	92.55	0.30	4.72	0.61	0.00
XY crack	0.17	0.06	1.07	94.23	1.53	1.13	1.81
ZY crack	0.26	2.52	7.09	1.63	87.35	1.10	0.05
ZX crack	0.36	2.69	2.23	1.92	1.87	90.93	0.00
multiple	0.00	0.00	0.05	4.85	0.00	0.00	95.10
